# Study and Microanalysis on the Effect of the Addition of Polypropylene Fibres on the Bending Strength and Carbonization Resistance of Manufactured Sand Concrete

**DOI:** 10.3390/polym15092139

**Published:** 2023-04-29

**Authors:** Yan Tan, Chong Ma, Ben Zhao, Wei Xiong, Xingxiang Chen, Jiangtao Yu

**Affiliations:** 1 College of Civil Engineering, Architecture and Environment, Hubei University of Technology, Wuhan 430068, China; 102000671@hbut.edu.cn (C.M.); 102010859@hbut.edu.cn (B.Z.); 101910600@hbut.edu.cn (X.C.); 2 Department of Architecture and Engineering, Wuhan City Polytechnic, Wuhan 430064, China; 02009026@whcp.edu.cn; 3 School of Civil Engineering, Tongji University, Shanghai 200092, China; yujiangtao@tongji.edu.cn

**Keywords:** polypropylene fibre, manufactured sand concrete, bending strength, carbonization resistance, carbonization depth prediction model, response surface model

## Abstract

To popularize the complete replacement of natural sand with manufactured sand, a study was performed to determine the effect of adding polypropylene fibres (PPFs) to increase the bending strength and carbonization resistance of manufactured sand concrete (MSC). A 2 × 3 factorial design with the content and length of PPF as variables was used to establish a carbonization depth prediction model and a response surface model (RSM). The phase composition and microstructure of polypropylene-fibre-reinforced manufactured sand concrete (PPF-MSC) were analysed using X-ray diffraction (XRD) and scanning electron microscopy (SEM). The results show the addition of PPF with different contents and lengths increases the bending strength of PPF-MSC to varying degrees, while reducing the carbonization depth and increasing the dynamic elastic modulus after 28 days of carbonization. The highest bending strength (6.12 MPa) and carbonization resistance of PPF-MSC are obtained by the addition of 1 kg/m^3^ of 12 mm PPF, while the carbonization depth and an increase in the dynamic elastic modulus after 28 days of carbonization are maintained at a minimum of 2.26% and 1.94 mm, respectively. A prediction model was established to obtain a formula for the PPF-MSC carbonization depth in terms of the content and length of PPF and the carbonization time. The following results were obtained from the RSM: compared to the PPF length, the PPF content has a larger impact on the PPF-MSC bending strength and a smaller impact on the PPF-MSC carbonization resistance; there is no significant interaction between the content and length of PPF; and the predicted and measured values are close, indicating that the model is highly reliable. A comparison of the XRD patterns and SEM micrographs of PPF-MSC and MSC after 28 days of carbonization show a lower peak intensity of CaCO_3_ in the pattern for the carbonized area for PPF-MSC than for MSC and considerably fewer surface pores and cracks in PPF-MSC than in MSC. These results indicate that the addition of PPF increases the compactness of MSC and creates an effective resistance to the erosion by water molecules and carbon dioxide (CO_2_), thus enhancing the bending strength and carbonization resistance of MSC.

## 1. Introduction

The escalating global environmental crisis has created a growing demand for sustainable building materials, making it crucial to design novel types of sustainable concrete [1,2,3]. Concrete is a typical inorganic composite material with widespread use in construction engineering [4]. Natural sand is the most commonly used fine aggregate in concrete, but natural sand resources are becoming increasingly scarce, with a global yield ranging from 32 to 50 billion tons [5]. Overmining of natural sand could negatively impact society and the environment, ultimately affecting the operation of freshwater ecosystems [6,7,8,9]. The average annual construction demand for sand in China exceeds 13 billion tons, of which the highest demand is for natural sand. Therefore, to prevent excessive mining of natural sand, the Chinese government has stipulated since 2002 that sand mining in the Yangtze River shall not exceed 50 million tons per year, which is far from sufficient to meet engineering construction needs. Manufactured sand is a sandstone material obtained using professional sand production equipment, such as crushers. Compared with natural sand, manufactured sand has sharper edges and corners, a higher preponderance of needle-like particles, a wider range of sources, and a lower price [10]. Manufactured sand particles have irregular shapes. The adhesive characteristics of manufactured sand can typically be improved by combination with cement or other materials. Manufactured sand has good construction performance, and the compression and tensile strengths of manufactured sand concrete (MSC) are comparable to those of natural sand concrete (NSC) [11]. Fostering the development of the manufactured sand and stone industry has been encouraged in China (see Guidance on Improving the Quality of Development in the Manufactured Sand and Stone Industry issued by the Ministry of Industry and Information Technology and other departments, as well as Guidance on Achieving Rigorous and Systematic Development of the Sand and Stone Industry jointly issued by 15 departments and units (including the National Development and Reform Commission)). Therefore, the replacement of natural sand by manufactured sand in industry appears to be inevitable.

Carbonization is one of the influencing factors for the durability of building structures. Carbonization, also known as concrete neutralization, is a term used to describe a failure mode in concrete. During the carbonization of concrete, carbon dioxide (CO_2_) in the air inside the numerous capillary pores in concrete reacts with free carbon hydroxide (Ca(OH)_2_) in concrete to generate calcium carbonate (CaCO_3_), causing loosening of the concrete structure and falling off of concrete pieces. Carbonization can reduce the alkalinity of concrete. When the carbonization depth exceeds the thickness of the concrete protection layer, the concrete can no longer protect steel bars against the action of water and air, leading to a series of problems, such as rusting, shrinkage cracking, and even disintegration of the gel structure in concrete [12,13]. Vijaya et al. [14] used scanning electron microscopy (SEM) to observe the microstructures of manufactured and natural sands and found that compared to natural sand, manufactured sand contains more needle-shaped and angular particles and has a rougher surface. Zhao et al. [15] prepared C30, C40, and C50 MSCs and studied the effect of long-term carbonization on the MSC performance. The carbonization characteristics of MSC and NSC with the same strength grades were found to be similar, i.e., carbonization was fast up to 28 days and then slowed. Ramesh et al. [16] reported similar strengths and corrosion resistances for MSC and NSC. Compared to NSC, MSC exhibits enhanced bonding between aggregate and cement paste, a more even stress distribution, and a higher flexural capacity. However, the angularity of manufactured sand particles does not promote compactness of the interfacial transition zone, decreasing the cracking load and accelerating crack propagation [17,18]. MSC has a smaller carbonization depth and a higher carbonization resistance than NSC [19]. Although MSC has a higher bending strength and carbonization resistance than NSC, poor cracking resistance makes MSC more prone to cracking than NSC, which accelerates erosion by water molecules and CO_2_, promotes carbonization reactions, and increases the vulnerability of the concrete protection layer to environmental attack, ultimately increasing the rusting of reinforcing steel.

Studies have shown that the addition of fibres can increase the carbonization resistance of concrete [20,21,22]. To strengthen the structure, carbon fibre can be used. However, the high price, difficulty in recycling, and lack of availability of carbon fibre are weaknesses [23]. Polypropylene fibre (PPF) offers the advantages of a low price and good crack resistance over steel, basalt, polyvinyl alcohol, and polyethylene fibres [24,25,26]. In particular, PPF can reduce the workability of concrete [27]. The addition of PPF to concrete results in the formation of a uniform random support system with a small fibre spacing that can effectively inhibit crack development and the formation of infiltration channels [28,29,30,31,32], reduce porosity, and densify the concrete microstructure [33,34], thereby increasing the compressive strength [35,36,37], tensile properties, and bending strength [38,39]. Maek et al. [40] investigated the mechanical properties of concrete with 0.5%, 1.0%, and 1.5% of two types of added regenerated PPF. The concrete with 1.0% PPF exhibited the best mechanical properties. Li et al. [41] used nuclear magnetic resonance and SEM to determine the effect of PPF on the concrete microstructure. As PPF has a high tensile strength, the addition of a small quantity of PPF considerably improves the deformation ability of concrete. However, the addition of PPF has little effect on the pore size and porosity of concrete, and the addition of excessive PPF increases the contact area between different materials and causes stress concentration in the fibres, increasing the number of destructive cracks in concrete and decreasing the carbonization resistance. The optimal content of added PPF was found to be approximately 0.6%. He et al. [42] showed that PPF addition can reduce the porosity of MSC and thereby densify the structure, where optimal MSC performance was achieved using 0.6 kg/m^3^ PPF.

Recent studies on polypropylene-fibre-reinforced manufactured sand concrete (PPF-MSC) have mainly focused on the material mechanical properties, and more research needs to be conducted on the durability of PPF-MSC. Therefore, in this study, natural sand in MSC was completely replaced with manufactured sand with different contents of PPF (of various lengths). The influence of the content and length of PPF on the bending strength and carbonization resistance of MSC was studied. The changes in the carbonization depth of MSC were used to establish a carbonization depth prediction model. The changes in the bending strength, dynamic elastic modulus, and carbonization depth of MSC were used to establish a response surface model (RSM). The phase composition and microstructure of PPF-MSC were determined using X-ray diffractometry (XRD) and SEM. The analysis of the bending strength and carbonization resistance of PPF-MSC performed in this study is highly relevant for practical applications.

## 2. Materials and Methods

### 2.1. Raw Materials

**Figure 1:**
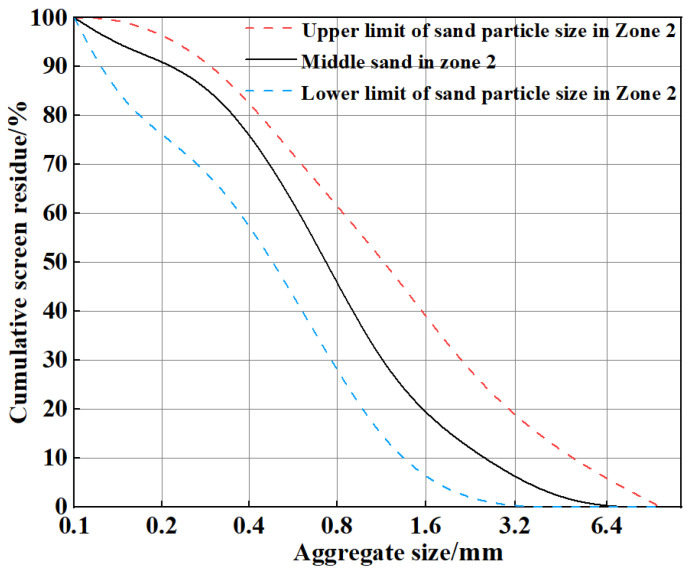
Cumulative weight of the screen residue of manufactured sand.

**Figure 2:**
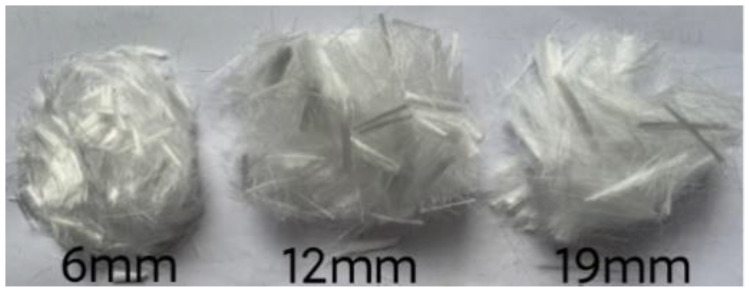
Polypropylene fibre (PPF).

Tests were performed on P·O 42.5 cement, a mineral admixture of Grade I fly ash, natural coarse aggregates with particle sizes ranging from 5 mm to 33 mm, and Zone II medium sand as manufactured sand. According to Sand for Construction (GB/T14684-2011), the megabyte (MB) value and stone powder content of the manufactured sand meet the requirements of Grade II manufactured sand. The cumulative screen residue and main performance indicators of the manufactured sand are shown in Figure 1 and Table 1, respectively. Figure 2 shows PPF with lengths of 6 mm, 12 mm, and 19 mm that was provided by the Direct Sales Department of the Changsha Huixiang Fibre Factory. The PPF performance indicators are shown in Table 2. A high-performance polycarboxylate superplasticizer made in China was used as a water-reducing agent.

### 2.2. Mix Proportion Design and Specimen Fabrication

The following contents and lengths of PPF were used to fabricate a concrete specimen with a design strength grade of C30 following the Specification for Mix Proportion Design of Ordinary Concrete (JGJ55-2011): 0.8 kg/m^3^, 1.0 kg/m^3^, and 1.2 kg/m^3^; 6 mm, 12 mm, and 19 mm. Table 3 shows the concrete mix proportions.

**Figure 3:**
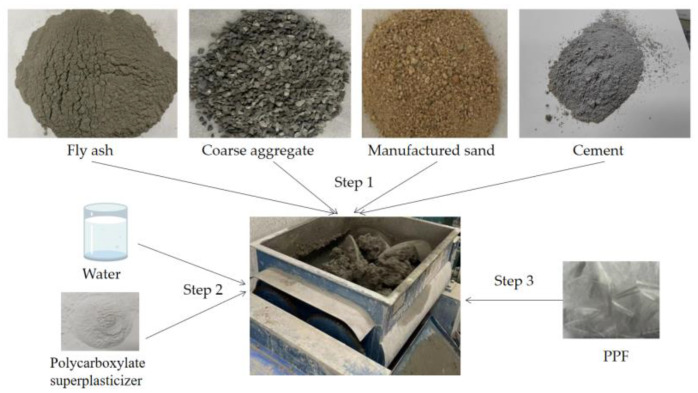
Schematic figure.

First, a single horizontal shaft forced concrete mixer (HJW-60) was used to mix coarse aggregate, fine aggregate, and cement for 60 s. Next, a polycarboxylate superplasticizer and water were added to the mixer, and the mixture was homogenized. Finally, PPF was added and evenly dispersed into the concrete matrix by 3 min of mixing. The schematic figure is shown in Figure 3. The mixture was compacted into a 100 mm × 100 mm × 400 mm specimen using a vibration table and demoulded after standard curing (24 h of curing at normal temperature for 28 days).

### 2.3. Test Scheme

Following the Standard for Test Methods of Long-term Performance and Durability of Ordinary Concrete (GB/T50082-2009), the test specimen was removed from the curing room two days before the carbonization test to be performed and dried for 48 h at 60 °C. The two opposite side surfaces of the specimen were left untreated, and the other surfaces were sealed with heated paraffin. Parallel lines were drawn on the side surfaces of the specimen along the length at 10 mm intervals for measuring the carbonization depth.

**Figure 4:**
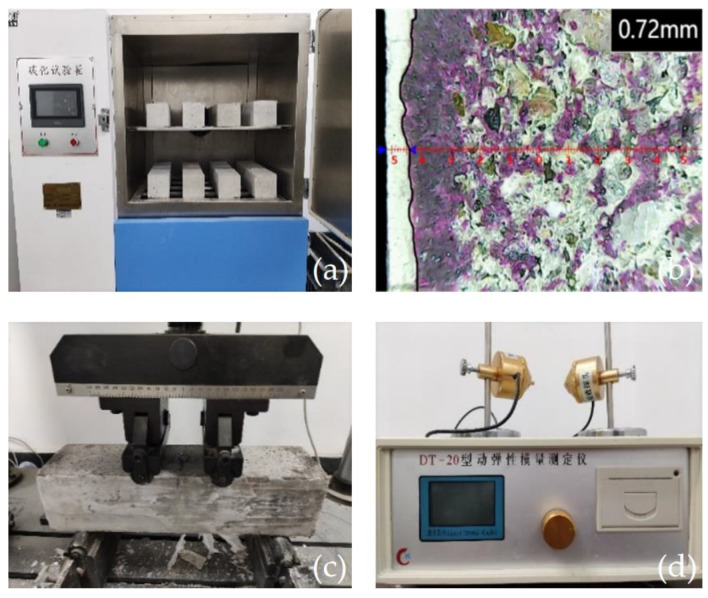
Test drawing: (**a**) carbonization test; (**b**) measurement of the carbonization depth; (**c**) bending strength test; and (**d**) dynamic elastic modulus tester.

Next, the test specimen was placed in a TH-B concrete carbonization test box, as shown in Figure 4a. The CO_2_ concentration was 20 ± 3%, the relative humidity was 70 ± 5%, and the temperature was 20 ± 5 °C. At 3, 7, 14, and 28 days after carbonization, the test specimen was removed from the test box and broken using a DYE-2000S compression testing machine. A specimen cross section was sprayed with a 1% phenolphthalein alcohol solution. A KS-105 wireless crack-width gauge was used to measure the carbonization depth as the distance from each point on both sides of the specimen to the purple boundary, as shown in Figure 4b. The average carbonization depth of concrete at different test ages was calculated using Formula (1):

(1) $$\bar{d_{t}}=\frac{1}{n}\sum_{i-1}^{n}d_{i}$$

where $\bar{d_{t}}$ denotes the average carbonization depth (mm) for the specimen after t days, *d_i_* denotes the carbonization depth (mm) measured for each of the two side surfaces, and *n* denotes the total number of measurement points on the two side surfaces. The test described above was performed on three specimens of each group, and the measurements were averaged.

An MTS compression testing machine was employed to measure the bending strength, as shown in Figure 4c. A DT-20 dynamic elastic modulus tester was used to measure the dynamic elastic modulus of the test specimen before and after 28 days of carbonization, as shown in Figure 4d.

## 3. Results and Discussion

### 3.1. Analysis of the Bending Strength

**Figure 5:**
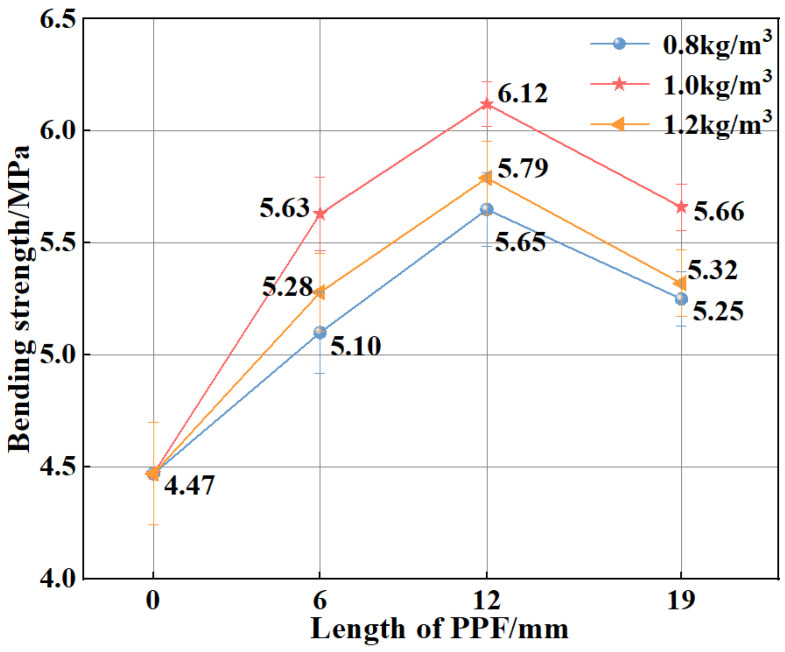
Bending strengths of MSC and PPF-MSC.

Figure 5 shows the bending strengths of MSC and PPF-MSC. PPF-MSC has a higher bending strength than MSC because of the ability of PPF to inhibit crack propagation, concentrate stress at the crack tip, and decrease the crack growth rate. The tight connection between PPF and the substrate also enhances the ability of PPF-MSC to withstand vertical loads. As the content and length of PPF increase, the bending strength of PPF-MSC first increases and then decreases. The maximum increase of 36.91% in the bending strength of MSC is obtained for PPF (C_1_L_12_)-MSC (which has a bending strength of 6.12 MPa). This result is obtained because PPF of a moderate length and content disperses effectively and uniformly in the concrete, and PPF has good bridging ability that enables a tight combination with the matrix, maximizing the increase in the MSC bending strength. An excessively high PPF content results in a narrow spacing between the fibres in the concrete, increasing the tendency of the fibres to knot and agglomerate. The PPF-MSC bending strength consequently decreases. When the fibre length is too large, the longer fibres cannot completely fill the internal pores of the concrete, thus reducing the compactness of the concrete, resulting in lower bending strength.

### 3.2. Analysis of Carbonization Resistance

**Figure 6:**
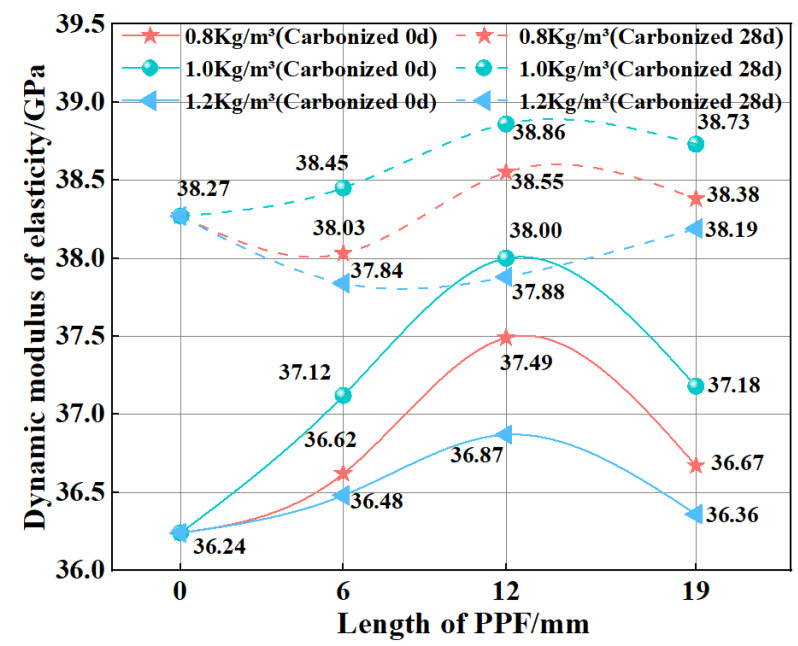
Dynamic elastic moduli of MSC and PPF-MSC before and after 28 days of carbonization.

Figure 6 shows the dynamic elastic moduli of MSC and PPF-MSC before and after 28 days of carbonization. The dynamic elastic modulus of the different uncarbonized PPF-MSC samples is higher than that of uncarbonized MSC to different degrees, indicating that PPF-MSC has a denser structure than MSC. As the content and length of PPF increase, the dynamic elastic modulus of PPF-MSC first increases and then decreases. The maximum increase (4.86%) in the dynamic elastic modulus of MSC is obtained for PPF (C_1_L_12_)-MSC (which has a dynamic elastic modulus of 38 GPa). Both the bending strength and dynamic elastic modulus of MSC and PPF-MSC increase after 28 days of carbonization. This result is obtained because CaCO_3_ generated by the carbonization reaction fills the small pores in the concrete surface to compactify the concrete structure, thereby increasing the dynamic elastic modulus. 

**Figure 7:**
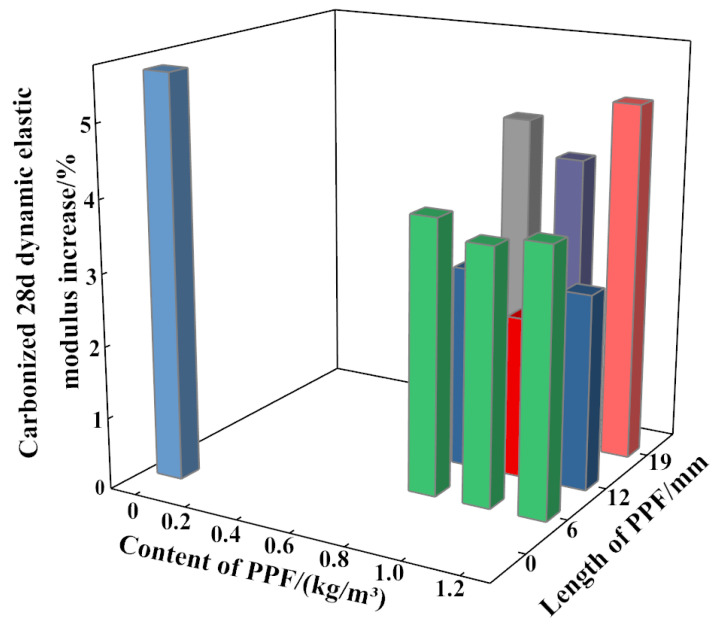
Increase in the dynamic elastic modulus of MSC and PPF-MSC before and after 28 days of carbonization.

Figure 7 shows the increases in the dynamic elastic moduli of both MSC and PPF-MSC after 28 days of carbonization. The increase in the dynamic elastic modulus is lower for PPF-MSC than MSC. As the content and length of PPF in PPF-MSC increase, the increase in the dynamic elastic modulus first decreases and then increases. The increase in the dynamic elastic modulus is smallest (2.26%) for PPF(C_1_L_12_)-MSC and largest (5.6%) for MSC. The increase in the dynamic elastic modulus of PPF(C_1_L_12_)-MSC is 59.64% lower than that of MSC. Carbonization can cause a decrease in the permeability and porosity of concrete, which is particularly significant for concrete with poor performance. Comparing the increases in the dynamic elastic modulus for MSC and PPF-MSC shows a larger decrease in the internal porosity after 28 days of carbonization for MSC than for PPF-MSC, indicating that carbonization has a larger impact on MSC than on PPF-MSC. There are two main reasons for this phenomenon. First, PPF can resist cracking and is randomly distributed in the concrete, thereby blocking the capillary channels and affecting the movement of water molecules. Consequently, the number of connected voids created by water seepage are effectively reduced, the formation of internal cracks in the concrete is hindered, and the propagation rate of CO_2_ in the concrete is decreased. Second, PPF can support aggregates, which effectively prevents segregation in concrete caused by aggregate settling. Consequently, the porosity is reduced and the pore structure is improved, providing effective resistance against the invasion of CO_2_ into the concrete and decreasing the carbonization rate.

**Figure 8:**
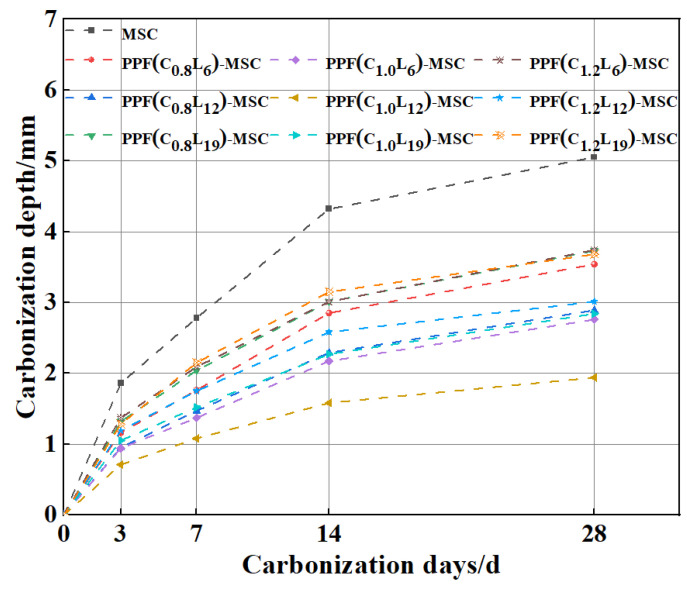
Variation in the carbonization depth with time for MSC and PPF-MSC.

Figure 8 shows that the carbonization depths of MSC and PPF-MSC gradually increase with time. The carbonization rate first increases and then decreases with time. The maximum change in the carbonization depth occurs from 7 to 14 days and gradually decreases from 14 to 28 days, indicating that the carbonization rate decreases. During the early stage of carbonization, CO_2_ enters the concrete and reacts rapidly with Ca(OH)_2_ on the surface, generating a large quantity of CaCO_3_. During the late stages of carbonization, the formation of a thin CaCO_3_ layer on the surface of the Ca(OH)_2_ crystal causes the carbonization rate to decrease. The quantity of carbonization products increases and fills the pores on the concrete surface, which blocks the invasion of CO_2_ and decreases the carbonization rate. As the CaCO_3_ product layer gradually thickens, contact between the uncarbonized part of the concrete and water is increasingly inhibited, making it difficult for H_2_CO_3_ to be generated by the invasion of CO_2_. Consequently, the rate of the carbonization reaction between Ca(OH)_2_ and the hydration product (C-S-H gel) decreases. Thus, the carbonization rate first increases and then decreases. However, the change in the carbonization depth of PPF-MSC during the early and late stages of carbonization is considerably lower than that of MSC, indicating that PPF addition decreases the carbonization rate of MSC. This result is obtained because PPF can both inhibit cracking and enhance interfacial effects, that is, the formation of a cement mortar fibre structure in the concrete creates many interfaces that effectively relax stress on the material. When a microcrack appears, an interface can effectively alleviate the stress at the crack tip to delay crack expansion [22], thereby effectively reducing erosion by CO_2_ and the carbonization depth.

**Figure 9:**
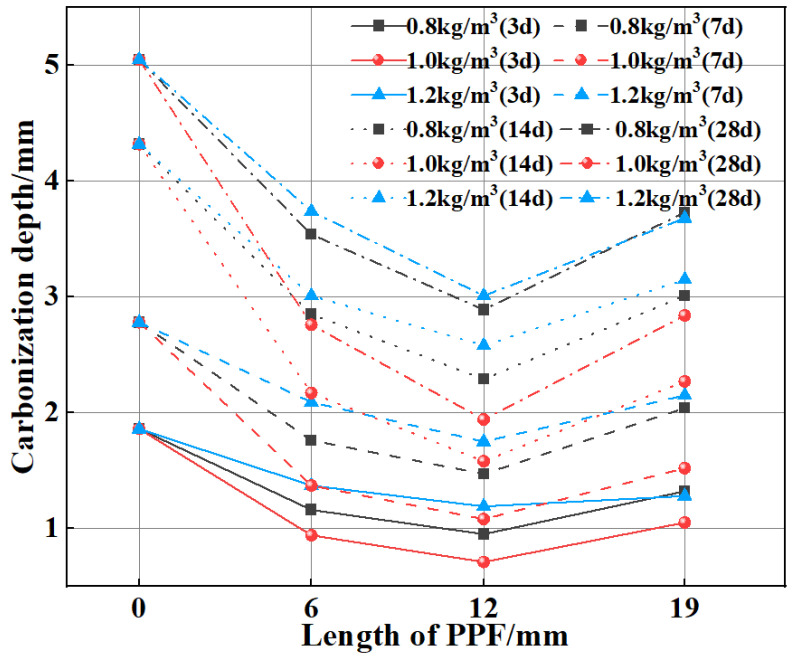
Variation in the carbonization depth with the length and content of PPF for MSC and PPF-MSC.

Figure 9 shows that the carbonization depths of MSC and PPF-MSC first increase and then decrease as the length and content of PPF increase. The carbonization depth after 28 days of carbonization reaches a minimum of 1.94 mm for PPF(C_1_L_12_)-MSC, which is 61.58% lower than the corresponding value for MSC. The average carbonization depth for the PPF-MSC specimens is 38.02% lower than that of MSC, indicating an improvement in the carbonization resistance of MSC.

### 3.3. Carbonization Depth Prediction Model

The assumptions that concrete is an isotropic continuous medium material and that steady-state diffusion theory can be used to simulate the diffusion process of CO_2_ are widely accepted by researchers. The carbonization depth is determined using the following formula:

(2) $$H=k\sqrt{t}$$

where *H* denotes the carbonization depth (mm), *k* denotes the carbonization coefficient, and *t* denotes the carbonization time (d).

**Figure 10:**
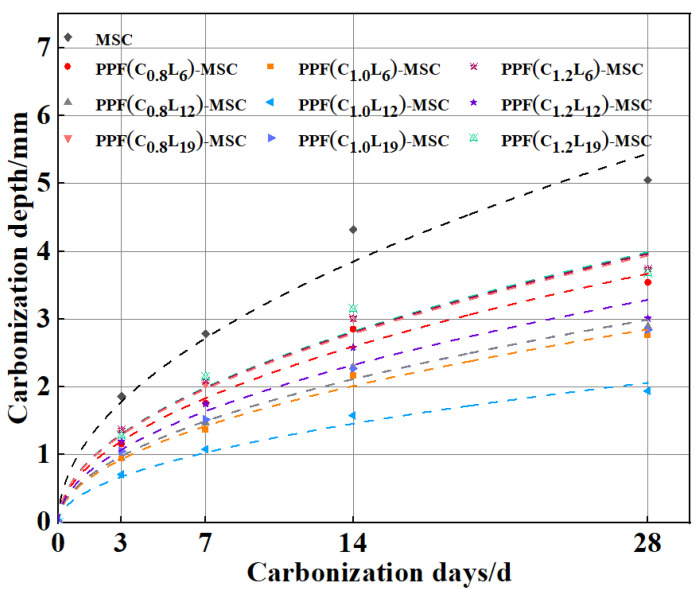
Fitting curve for the carbonization model.

The data for the carbonization depth with time were fitted. The corresponding formula and coefficient of determination (R^2^) for the fit are shown in Table 4, and the fitted curve is shown in Figure 10. The R^2^ value is >0.95 for all the fitting results, indicating a good fit. MSC has the largest carbonization coefficient and the lowest resistance to carbonization. As the content and length of PPF increase, the carbonization coefficient of PPF-MSC first decreases and then increases. The lowest carbonization coefficient is obtained for PPF(C_1_L_12_)-MSC, which indicates that the highest carbonization resistance of PPF-MSC is obtained for a PPF content of 1 kg/m^3^ and a PPF length of 12 mm.

**Figure 11:**
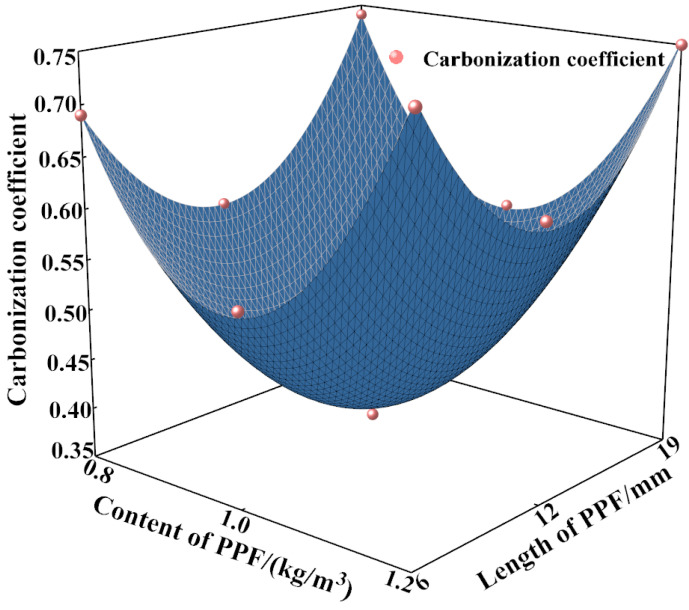
Variation in the carbonization coefficient with the content and length of PPF.

Figure 11 shows the results for a three-dimensional (3D) fit for the correlation of the carbonization coefficient shown in Table 4 with the content (*x*) and length (*y*) of PPF, where

*K* = (−9.28*x* − 0.07*y* + 4.75*x*^2^ + 0.003*y*^2^ − 0.01*xy* + 5.44) (3)

R^2^ = 0.993

Combining Equations (2) and (3) yields a prediction formula for the carbonization depth in terms of the PPF content, PPF length, and carbonization time:

(4) $$H=(-9.28x-0.07y+4.75x^{2}\;+0.003y^{2}-0.01\mathrm{xy}+5.4)\sqrt{t}$$

### 3.4. Establishment and Analysis of an RSM

The RSM is a commonly used method for multi-factor experimental design that has been widely applied to optimize industrial formulas and production conditions. However, the RSM has seldom been applied to concrete. An experiment was performed with the PPF content as Factor A and the PPF length as Factor B using Design Expert 10 to establish a multiple regression equation and regression model. The bending strength of PPF-MSC, the carbonization depth, and the increase in the dynamic elastic modulus after 28 days of carbonization were taken as the response values. The RSM was used to analyse the bending strength and carbonization resistance of PPF-MSC. To reduce errors, four sets of repeated experiments were designed for the RSM. That is, four sets of test blocks were reproduced according to Table 3 for the experiment. The experimental results are shown in Table 5.

The multiple regression equations and variance analysis of the regression model are shown in Table 6 and Table 7, respectively. The *p* values are <0.01 for the model of the PPF bending strength, as well as for the models of the carbonization depth and the increase in the dynamic elastic modulus after 28 days of carbonization, demonstrating that the models are extremely significant. The *p* values for Factor A in the model are 0.013 (<0.05), 0.626 (>0.05), and 0.049 (<0.05), indicating that the PPF content significantly affects the models for the bending strength and carbonization depth after 28 days of carbonization but does not significantly affect the model for the increase in the dynamic elastic modulus after 28 days of carbonization. The *p* values for Factor B in the model are 0.102 (>0.05), 0.0002 (<0.05), and 0.026 (<0.05), indicating that the PPF content significantly affects the models for the increase in the dynamic elastic modulus and carbonization depth after 28 days of carbonization but has an insignificant effect on the model for the bending strength. This result demonstrates that compared to the PFF length, the PPF content has a larger impact on the bending strength of PPF-MSC but a smaller impact on the carbonization resistance of PPF-MSC. The AB values in the model are 0.285 (>0.05), 0.136 (>0.05), and 0.397 (>0.05), showing an insignificant interaction between the content and length of PPF.

**Figure 12:**
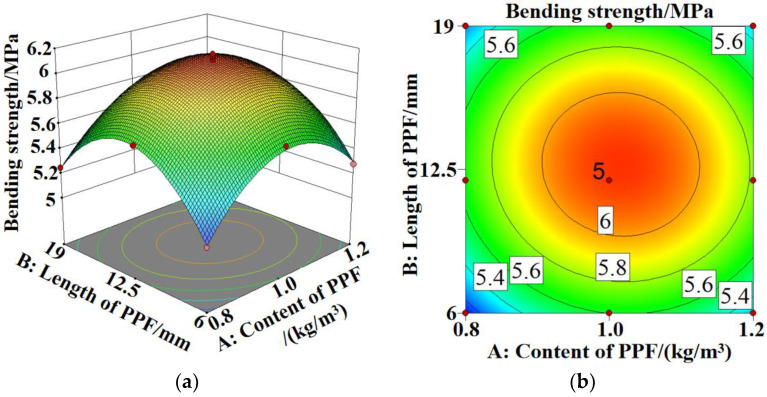
Bending strength model of PPF-MSC: (**a**) 3D response surface; (**b**) contour map.

**Figure 13:**
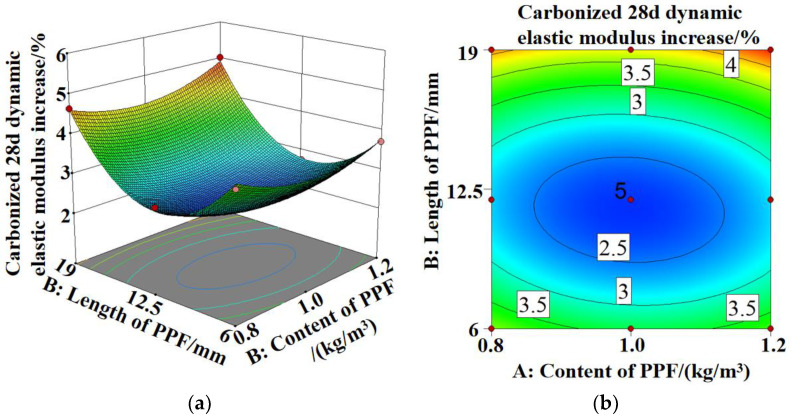
Increase in the dynamic elastic modulus of PPF-MSC after 28 days of carbonization: (**a**) 3D response surface; (**b**) contour map.

**Figure 14:**
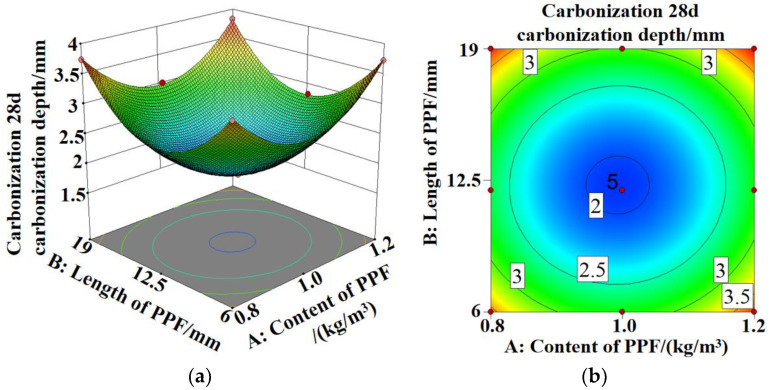
Carbonization depth model of PPF-MSC after 28 days of carbonization: (**a**) 3D response surface; (**b**) contour map.

Figure 12, Figure 13 and Figure 14a,b show the 3D response surface and contour maps for the bending strength of PPF-MSC, the increase in the dynamic elastic modulus after 28 days of carbonization, and the carbonization depth after 28 days of carbonization, respectively.

In Figure 12a,b the 3D surface is steeper and the contour lines are more dense along the direction of the PPF content than along the direction of the PPF length. This result indicates that the PPF content has a larger impact on the bending strength than the PPF length. In Figure 13a,b and Figure 14a,b, the 3D surface is steeper and the contour lines are denser along the direction of the PPF length than along the direction of the PPF content. This result indicates that the PPF length has a larger impact on the increase in the dynamic elastic modulus and the carbonization depth after 28 days of carbonization than the PPF content. The more elliptical the contour lines are, the more significant the interaction between Factors A and B is. However, all the contour maps shown in Figure 12b, Figure 13b and Figure 14b are fairly circular. This result demonstrates an insignificant interaction between the length and content of PPF, which is consistent with the results for the variance analysis of the regression model.

**Figure 15:**
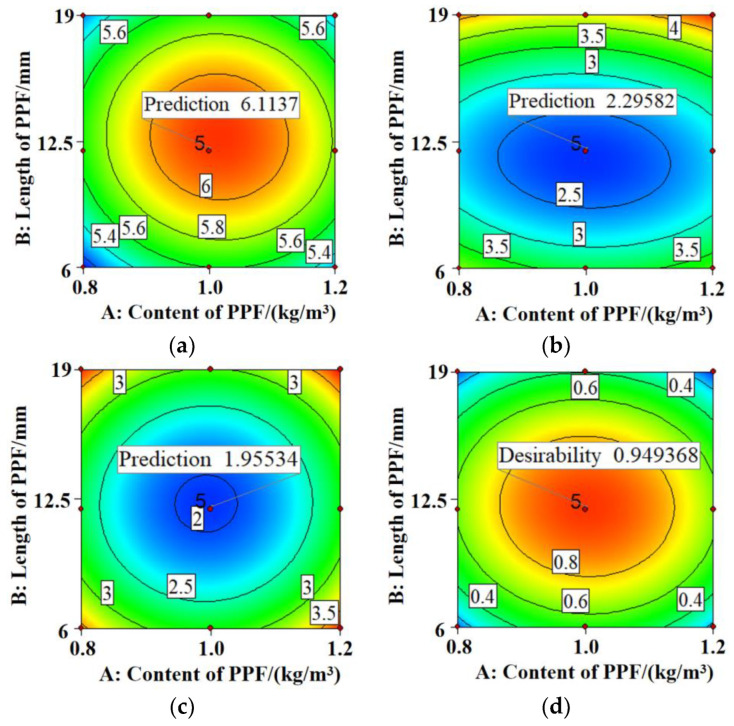
RSM prediction: (**a**) bending strength; (**b**) increase in the dynamic elastic modulus; (**c**) carbonization depth; and (**d**) desirability.

The RSM prediction results are shown in Figure 15. At a PPF content of 1.002 kg/m^3^ and a PPF length of 12.152 mm, the PPF bending strength reaches a maximum of 6.114 MPa, and the increase in the dynamic elastic modulus and carbonization depth after 28 days of carbonization decrease to a minimum of 2.296% and 1.955 mm, respectively. At this point, PPF-MSC exhibits the highest bending strength and carbonization resistance and the model prediction is close to the measured value with a reliability of 0.949, indicating high accuracy for the RSM prediction.

## 4. Microanalysis

### 4.1. Phase Composition Testing and Analysis

**Figure 16:**
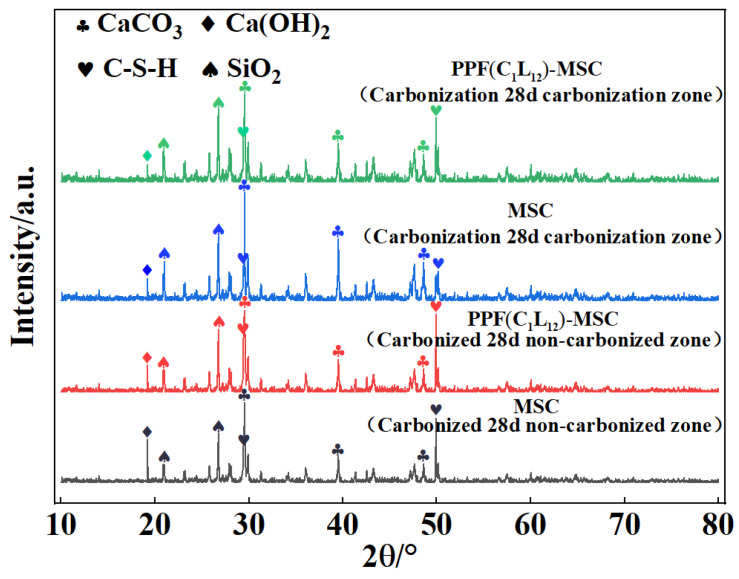
XRD patterns showing the phase compositions for carbonized and non-carbonized areas of MSC and PPF(C_1_L_12_)-MSC after 28 days of carbonization.

The phase compositions of the carbonized and non-carbonized areas of MSC and PPF(C_1_L_12_)-MSC (which was found to exhibit the highest resistance to carbonization) after 28 days of carbonization were determined using a German Bruker D8 Advance 25 XRD. The obtained XRD patterns presented in Figure 16 show that both the carbonized and non-carbonized areas of MSC and PPF(C_1_L_12_)-MSC contain CaCO_3_, Ca(OH)_2_, C-S-H, and SiO_2_. In particular, in the diffraction patterns for the carbonized areas of MSC and PPF(C_1_L_12_)-MSC, the peak intensities are higher for CaCO_3_ and SiO_2_ and lower for Ca(OH)_2_ and C-S-H than those for the non-carbonized areas. However, compared to the peak intensities in the PPF(C_1_L_12_)-MSC patterns, the peak intensities in the MSC patterns are higher for CaCO_3_ and SiO_2_ and lower for Ca(OH)_2_ and C-S-H. This result indicates that during CO_2_ corrosion, more Ca(OH)_2_ and C-S-H are consumed to react with CO_2_ and more CaCO_3_ and SiO_2_ are produced in MSC than in PPF(C_1_L_12_)-MSC. The addition of PPF can effectively slow MSC carbonization, thus improving the resistance of MSC to carbonization.

### 4.2. Microstructure Analysis

A Czech TESCAN MIRA LMS SEM was used to determine the microstructure of MSC and PPF (C_1_L_12_)-MSC (which was found to exhibit the highest bending strength and carbonization resistance). Two observation areas, labelled A and B, were randomly selected from the two specimens.

**Figure 17:**
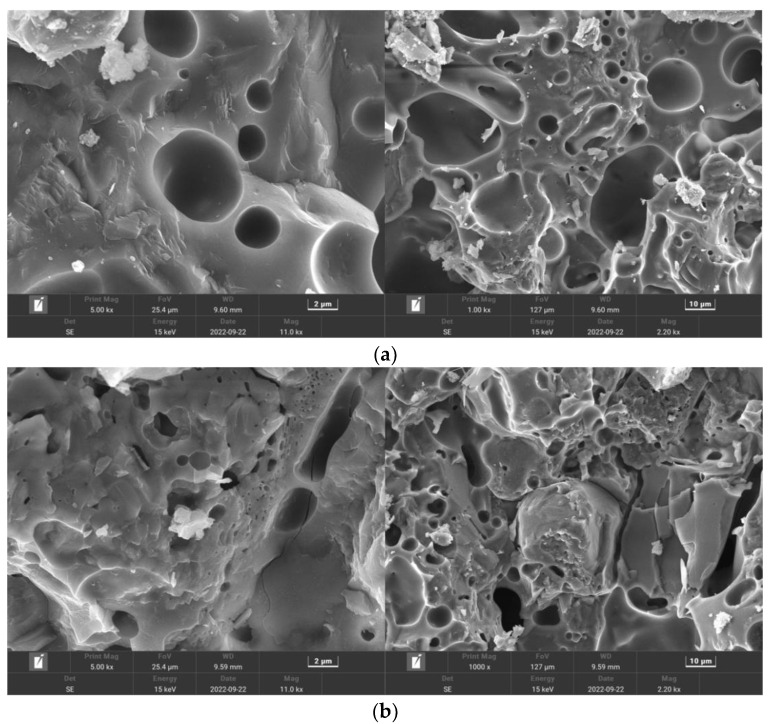
MSC microstructure: (**a**) Area A; (**b**) Area B.

**Figure 18:**
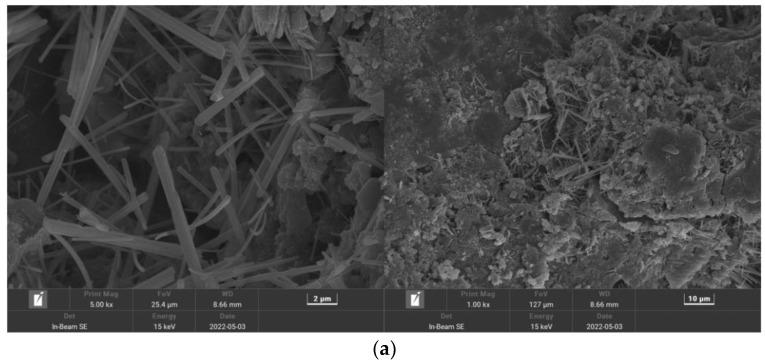

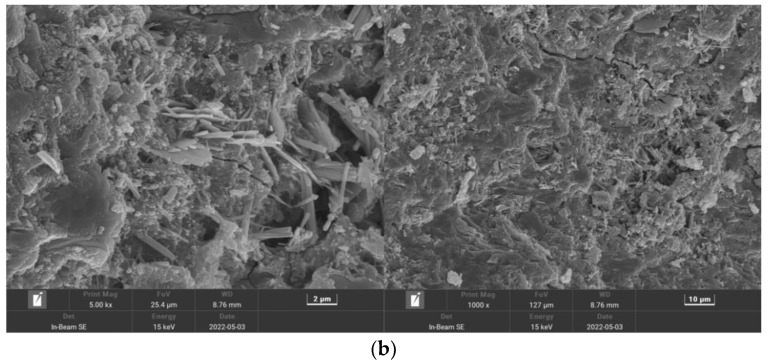
PPF (C_1_L_12_)—MSC microstructure: (**a**) Area A; (**b**) Area B.

**Figure 19:**
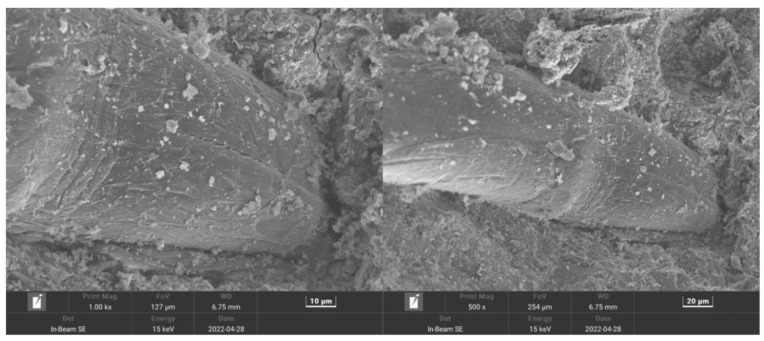
Microstructure of the PPF (C_1_L_12_)—MSC interface.

Figure 17 shows the MSC microstructure, where the matrix surface appears rough with some cracks and numerous large pores. Figure 18 shows the PPF (C_1_L_12_)-MSC microstructure, where the matrix surface appears relatively smooth and flat with few pores and cracks. Figure 19 shows the microstructure of the interface between PPF and the matrix interface. The excellent bridging ability of PPF results in a tight connection between PPF and the matrix, which considerably increases the matrix density over that of MSC. As a result, erosion of the internal material of the matrix by water and CO_2_ is reduced, and the carbonization reaction is slowed. Therefore, PPF addition to MSC can improve the pore structure and density while enhancing the bending strength and carbonization resistance.

## 5. Conclusions

(1) In general, the addition of PPF to MSC results in an increase in the bending strength, while reducing the increase in the dynamic elastic modulus and carbonization depth after 28 days of carbonization. Thus, PPF addition enhances the bending strength and carbonization resistance of MSC.

(2) The maximum enhancement of the bending strength of MSC (to 6.12 MPa) is obtained by the addition of 1 kg/m^3^ of 12 mm long PPF to MSC, whereas the increase in the dynamic elastic modulus and carbonization depth after 28 days of carbonization are maintained at a minimum of 2.26% and 1.94 mm, respectively, i.e., the highest bending strength and carbonization resistance of MSC are obtained.

(3) A prediction model for the carbonization depth is established to obtain a formula for predicting the MSC carbonization depth using the PPF content, PPF length, and carbonization time: *H* = (−9.28*x* − 0.07*y* + 4.75*x*^2^ + 0.003*y*^2^ − 0.01*xy* + 5.44)$\;\sqrt{t}$. An RSM shows that the PPF length has a larger influence on the bending strength of MSC and a smaller influence on the carbonization resistance of MSC than the PPF content and that the interaction between the content and length of PPF is not significant. Excellent agreement between the predicted and measured results indicates that the model is reliable.

(4) XRD and SEM results show that after 28 days of carbonization, the diffraction peak intensity in the pattern for the carbonized area of PPF-MSC is lower for CaCO_3_ and higher for C-S-H than in that of MSC. The PPF-MSC matrix is smoother and denser with fewer pores and cracks than MSC.

## Figures and Tables

**Table 1:** Main performance indicators of manufactured sand.

Fine Aggregate	Apparent Density (kg/m^3^)	Fineness	Bulk Density (kg/m^3^)	Silt Content (%)
Manufactured sand	2610	2.6~2.8	1500	1.8

**Table 2:** PPF performance indicators.

Type	Density (g/cm^3^)	Elasticity Modulus (MPa)	Breaking Strength (MPa)	Elongation at Break (%)	Melting Point (°C)	Ignition Point (°C)	Acid and Alkali Resistance
Fascicular	0.91	>4500	450	25 ± 5	165–175	590	Strong

**Table 3:** Concrete mix proportions.

No.	Fibre Content (kg/m^3^)	Fibre Length (mm)	Consumption of Materials (kg/m^3^)					
			Cement	Fly Ash	Manufactured Sand	Gravel	Water	Water Reducer
MSC	0	0	398	80	763	920	200	4.71
PPF(C_0.8_L_6_)-MSC	0.8	6	398	80	763	920	200	4.71
PPF(C_0.8_L_12_)-MSC	0.8	12	398	80	763	920	200	4.71
PPF(C_0.8_L_19_)-MSC	0.8	19	398	80	763	920	200	4.71
PPF(C_1_L_6_)-MSC	1	6	398	80	763	920	200	4.71
PPF(C_1_L_12_)-MSC	1	12	398	80	763	920	200	4.71
PPF(C_1_L_19_)-MSC	1	19	398	80	763	920	200	4.71
PPF(C_1.2_L_6_)-MSC	1.2	6	398	80	763	920	200	4.71
PPF(C_1.2_L_12_)-MSC	1.2	12	398	80	763	920	200	4.71
PPF(C_1.2_L_19_)-MSC	1.2	19	398	80	763	920	200	4.71

Note: MSC denotes MSC with no PPF, and PPF(C_0.8_L_6_)-MSC denotes MSC with 0.8 kg/m^3^ of 6-mm-long PPF.

**Table 4:** Fitting results for the relationship between the carbonization depth and time.

	Fitting Formula	R^2^
MSC	*H* = 1.03$\sqrt{t}$	0.976
PPF(C_0.8_L_6_)-MSC	*H* = 0.69$\sqrt{t}$	0.989
PPF(C_0.8_L_12_)-MSC	*H* = 0.57$\sqrt{t}$	0.992
PPF(C_0.8_L_19_)-MSC	*H* = 0.74$\sqrt{t}$	0.988
PPF(C_1_L_6_)-MSC	*H* = 0.54$\sqrt{t}$	0.992
PPF(C_1_L_12_)-MSC	*H* = 0.39$\sqrt{t}$	0.985
PPF(C_1_L_19_)-MSC	*H* = 0.56$\sqrt{t}$	0.989
PPF(C_1.2_L_6_)-MSC	*H* = 0.75$\sqrt{t}$	0.987
PPF(C_1.2_L_12_)-MSC	*H* = 0.62$\sqrt{t}$	0.971
PPF(C_1.2_L_19_)-MSC	*H* = 0.75$\sqrt{t}$	0.974

**Table 5:** RSM design results.

No.	PPF Content (kg/m^3^)	PPF Length (mm)	Bending Strength (MPa)	Increase in the Dynamic Elastic Modulus after 28 Days of Carbonization (%)	Carbonization Depth after 28 Days of Carbonization (mm)
1	0.8	6	5.1	3.84	3.54
2	1	6	5.63	3.58	2.89
3	1.2	6	5.28	3.72	3.73
4	0.8	12	5.65	2.83	2.76
5	1	12	6.12	2.15	2.03
6	1.2	12	5.79	2.74	2.84
7	0.8	19	5.25	4.65	3.74
8	1	19	5.66	4.17	3.01
9	1.2	19	5.32	5.03	3.82
10	1	12	6.15	2.26	1.94
11	1	12	6.08	2.45	1.86
12	1	12	6.17	2.16	1.97
13	1	12	6.02	2.38	1.91

**Table 6:** Multiple regression equations.

Response Value	Multiple Regression Equation
Bending strength	y_1_ = 6.12 + 0.064A + 0.037B − 0.028AB − 0.4A^2^ − 0.48B^2^
Increase in the dynamic elastic modulus	y_2_ = 2.32 + 0.032A + 0.45B + 0.13AB + 0.47A^2^ + 1.54B^2^
Carbonization depth	y_3_ = 1.96 + 0.058A + 0.068B − 0.027AB + 0.81A^2^ + 0.96B^2^

**Table 7:** Variance analysis of the regression model.

Source of Variance	Sum of Squares	Degrees of Freedom (DOF)	Mean Square	F Value	*p* Value
Bending strength model	1.720	5	0.346	151.47	<0.0001 **
A	0.025	1	0.025	10.85	0.013 *
B	0.008	1	0.008	3.53	0.102
AB	0.003	1	0.003	1.34	0.285
Model for the increase in the dynamic elastic modulus	11.70	5	2.34	101.70	<0.0001 **
A	0.006	1	0.006	0.26	0.626
B	1.22	1	1.22	53.21	0.0002 **
AB	0.065	1	0.065	2.84	0.136
Model for the carbonization depth	7.05	5	1.41	401.13	<0.0001 **
A	0.020	1	0.020	5.66	0.049 *
B	0.028	1	0.028	7.97	0.026 *
AB	0.03	1	0.003	0.81	0.397

Note: “*” denotes a significant difference (*p* < 0.05); “**” denotes an extremely significant difference (*p* < 0.01).

## Data Availability

The data presented in this study are available from the corresponding author upon reasonable request.
